# Prevalence of diffuse idiopathic skeletal hyperostosis and association with coronary artery calcifications in Slovenia

**DOI:** 10.2478/raon-2025-0008

**Published:** 2025-02-27

**Authors:** Vesna Lesjak, Timea Hebar, Maja Pirnat

**Affiliations:** 1 Radiology Department, University Medical Centre Maribor, Maribor, Slovenia; 2 Medical Faculty, University of Maribor, Maribor, Slovenia

**Keywords:** diffuse idiopathic skeletal hyperostosis, coronary artery calcification, epicardial adipose tissue, metabolic syndrome, body mass index, coronary artery disease

## Abstract

**Background:**

The aim of this study was to analyze the epidemiological aspects of diffuse idiopathic skeletal hyperostosis (DISH) patients in Slovenia, to evaluate the relationship between coronary CT angiography (CCTA)-derived epicardial adipose tissue (EAT) density and coronary artery calcifications (CAC) in patients with and without DISH, and study influencing factors of these parameters.

**Patients and methods:**

The research comprised patients referred for CCTA due to a clinical suspicion of coronary artery disease. DISH, CAC score and EAT attenuation were quantified using non-contrast imaging. Diagnosis of DISH was based on Resnick criteria. The CCTA was assessed for the presence of obstructive coronary artery disease (CAD). The association between DISH and the extent of CAC was explored, using correlation analysis and multivariate regression.

**Results:**

The study cohort included 219 participants. The prevalence of DISH was 7.8%. In univariate logistic regression, body mass index (BMI) (odds ratio [OR] 1.133, p = 0,005), age (OR 1.055, p = 0,032) and diabetes (OR 3.840, p = 0,015) were significantly associated with the condition. However, this association did not persist on multinomial multivariate analysis, but gender, age, hypertension and EAT attenuation were found to be significantly associated with the increasing CAC strata.

**Conclusions:**

The prevalence of DISH found is comparable with prior literature. There was no independent relationship between the prevalence of DISH and CAC. Our data point to a more nuanced and perhaps non-causal link between coronary artery disease and DISH.

## Introduction

Diffuse idiopathic skeletal hyperostosis (DISH) is a systemic condition, originally described in 1950 by Forestier and Querol.^1^ In 1976 most commonly used criteria to diagnose DISH were introduced by Resnick and Niwayama.^2^ New bone formation, partially in entheses, is the condition’s defining feature.^3^ It is known that DISH affects more men than women, and its incidence rises with age.^4^ Prevalence in Asian countries varies between 3.8% and 27.0%, in the USA between 7.7% and 13.2%, and in Italy 12.8%.^5,6^ The etiology of DISH is not utterly understood. The disorder is linked to metabolic syndrome and its components, including diabetes, obesity, and hypertension^7,8^, associations were reported with large waist circumference, cardiomegaly, hyperinsulinaemia, dyslipidaemia and hyperuricaemia.^3^ DISH is associated with increased calcifications in coronary arteries^3^, and also in thoracic^9^ and abdominal aorta.^10^ The risk of myocardial infarction is considerably higher in DISH patients.^11^

Between the myocardium and the visceral pericardium is a visceral fat deposit called epicardial adipose tissue (EAT).^12^ It surrounds the heart and coronary arteries, being vascularized by branches of the coronary arteries.^12,13^ EAT is metabolically active, has a thermogenic role, secretes cytokines with pro- and anti-atherosclerotic qualities, and is hypothesized to defend against mechanical injuries to the heart and coronary vessels.^14^ It is thought to have a role in the onset of atherosclerosis, although it is unclear whether systemic processes or paracrine effects of EAT directly contribute to the development of atherosclerosis.^15^ The research has shown abundant evidence of the correlation between EAT volume and cardiovascular risk factors, coronary artery calcification and major adverse cardiac events.^16^ There has been recently increased interest in EAT attenuation as a marker of risk.^17^ In some studies a lower EAT attenuation on non-contrast enhanced cardiac CT scans has been linked to the risk of future events18, whereas in other studies a higher EAT attenuation has been linked to an increased risk.^19^

Up until recently, vascular calcification was thought to be an inevitable result of aging, and the development of coronary artery calcification (CAC) was thought to be a passive process. The development of CAC is now recognized as an active pathogenic process.

The common feature of atherosclerosis - ectopic bone production is known as the cause of coronary artery calcification, and new bone formation being the defining feature of DISH led us to hypothesize that arterial calcification and the occurrence of DISH are strongly correlated.

To our knowledge, the prevalence of DISH in the Slovenian population has not been evaluated. Based on this framework, the objective of our study is to analyze the epidemiological aspects of DISH patients in Slovenia, to evaluate the relationship between coronary CT angiography (CCTA)- derived EAT density and CAC in patients with and without DISH, and study influencing factors of these parameters.

## Patients and methods

This cross-sectional study was conducted at the Department of Radiology, University Medical Centre Maribor. This study was conducted with approval of local ethics committee (UKC-MB-KME-24/21) and performed accordingly to the Declaration of Helsinki. All participants gave written informed consent.

### Study protocol

Between January 2022 and January 2024, adult patients referred for CCTA were included in the study. Participants responded to questionnaires assessing socio-demographic information, lifestyle and health-related factors, which contains self-reported information on age, gender, weight status, chronic diseases, smoking and physical activity. Exclusion criteria were age < 18 years, known malignancy and prior coronary artery bypass surgery. A total of 219 cases were included in the study.

### Body mass index (BMI)

We calculated the BMI by using self-reported height and weight following the formula: weight (kg) divided by height (m) squared. According to WHO standards, BMI was categorized into underweight (BMI < 18.5), normal (18,5–24.99), overweight (BMI ≥ 25) and obese (BMI ≥ 30).^20^

### CT acquisition protocol

All examinations were performed on Somatom Drive CT scanner (Siemens Medical Solutions, Erlengen, Germany). Noncontrast, non-gated CT scan was performed to measure the Agatson coronary artery calcification score (CACS), as described previously.^21^ The sum of the individual lesion scores from the four vessels; left main (LM), left anterior descending (LAD), circumflex (LCX), and right coronary artery (RCA) produced the total coronary calcium score. The Agatson Units were classified into four categories: 0, > 0 and < 100, 100–400, and > 400. These categories represent no, mild/minimal, moderate, and substantial plaque burden. In the same way the calcium score (Agatson) was measured for proximal thoracic aorta (from aortic root to the first branch of the aortic arch), aortic root and ascending aorta.

The EAT attenuation was measured on the same axial images used for CACS. Epicardial adipose tissue Hounsfield units (HU) were measured using regions of interest (ROI) near the proximal part of RCA, between the right atrium and right ventricular outflow tract, as previously described.^15^

Hepatic and splenic HU attenuation values were quantified by placing two ROI in the liver and one in spleen, in the same axial slice. Liver to spleen ratio was calculated by dividing the mean liver attenuation by the splenic HU. Nonalcoholic fatty liver disease (NAFLD) was defined as liver to spleen ratio < 1 and/or mean liver attenuation < 40 HU.^22^

A retrospective ECG-gated CCTA examination was done in all participants, to assess coronary artery disease (CAD). CCTA datasets were transferred to a workstation (Syngo.via VB10. Siemens Healthcare, Forchheim, Germany), and coronary arteries were evaluated for the presence of obstructive CAD (defined as at least one lesion causing the stenosis of lumen ≥ 50%). CCTA images were reconstructed with a slice thickness of 0.6 mm. The CT studies were evaluated by radiologists having more than five years of experience in cardiac imaging.

### Diffuse idiopathic skeletal hyperostosis (DISH)

Resnick classification criteria were used to define DISH: the presence of flowing bridging ossification of at least four contiguous vertebrae, (relative) preservation of the intervertebral disc height and the absence of apophyseal joint bony ankylosis, as described. The prevalence of DISH was diagnosed by a single musculoskeletal radiologist evaluating CT images.

### Metabolic syndrome

The metabolic syndrome (MetS) was defined according to the International Diabetes Federation (IDF) definition^23^: BMI greater than 30 kg/m^2^ (in this case the central obesity can be assumed and waist circumference measure is not necessary) plus any two of the four factors: 1 raised triglycerides (≥ 1.7 mmol/l) or specific treatment for this abnormality, 2 reduced HDL cholesterol (< 1.03 mmol/l in males or < 1.29 mmol/l in females) or specific treatment, 3 raised blood pressure (systolic BP ≥ 130 or diastolic ≥ 85 mmHg) or treatment for diagnosed hypertension, and 4 raised fasting plasma glucose (≥ 5.6 mmol/l) or previously diagnosed diabetes type 2.

### Covariates

Additional data were collected: age in years, sex (male, female), smoking behavior (current smoker yes/no) and physical activity (days per week). The presence of hypertension, diabetes mellitus type 2 and hypercholesterolemia was established by the question ‘Have you had these condition?’ and/or the self-reported usage of antihypertensive drugs, glucose lowering and lipid lowering drugs. Other chronic health conditions included angina pectoris, and previous myocardial infarction.

### Statistical analysis

All continuous variables were tested for normal distribution (Shapiro-Wilk test). Normally distributed variables are given as means and standard deviations (SD), non-normally distributed variables are given as median (interquartile range [Q1–Q3]) and categorical variables are presented as numbers and percentages (%). Comparisons of demographic characteristics and potential covariates between the DISH and no DISH groups were conducted using Mann-Whitney U test and independent sample t-test for continuous variables, and Chi-square test for categorical variables. Group-wise comparisons were performed with the Kruskal-Wallis test. Independent sample t-test, Pearson or Spearman rank correlations were calculated to determine the relationships between EAT attenuation and risk factors. We also evaluated the relationship between EAT attenuation and CT parameters using multivariable linear regression analyses. To determine the association between the presence of DISH, EAT and CAC, univariate and multivariate logistic regression analyses were performed. The models included DISH status (present or absent) as dependent factor and age, gender, BMI, eight, smoking status, diabetes, hypertension and hypercholesterolemia as independent variables. A multivariate multinomial logistic regression was performed with CAC categories (> 0 and < 100, 100–400, > 400) as independent factor and CACS = 0 as reference category and DISH status as dependent factor. Multivariate analyses were done in a stepwise backward elimination based on a p-value < 0,10. We analyzed the prevalence of DISH and CACS in the relation to the amount of risk factors (diabetes, BMI > 30, hypertension, hypercholesterolemia) present. Comparisons between the DISH and no DISH groups were conducted using Chi-square test. All statistical analyses were performed using the SPSS 29.0 software package (IBM, Armonk, NY, USA). All tests were 2-sided and a ‘P’ value of less than 0.05 was considered statistically significant.

## Results

A total of 219 participants were included in the study. The overall prevalence of DISH was 7.8%. The prevalence of DISH was about twice as high in males than in females (10.4% *νs*. 4.8%). The characteristics of the demographics and cardiovascular risk factors of participants with and without DISH are shown in Table 1. Compared to patients without DISH, those with DISH were significantly older (67.3 *νs*. 60.5 years). 42.6% of subjects were obese (45.5% men and 39.4% women). Among subjects with DISH, 68.8% were obese, compared to 40.4% of patients without DISH. NAFLD was present in 26.3% of participants; in 29.4 % of patients with DISH and in 26.0% of patients without DISH. Metabolic syndrome was present in 15.4% of participants, in subjects with DISH in 43.8%, compared to 13.0% of subjects without DISH.

**TABLE 1.** Characteristics of the cohort

	DISH	no DISH	p-value
Age (years), mean (SD)	67.3 ± 10.1	60.5 ± 12.2	**0.029**
Gender (f/m), N	5/12	99/103	0.120
Weight (kg), mean (SD)	96.6 ± 20.3	84.5 ± 17.5	**0.008**
Height (cm), mean (SD)	170.9 ± 6.5	171.0 ± 9.7	0.980
BMI (kg/m^2^), mean (SD)	32.8 ± 7.2	28.9 ± 5.3	**0.011**
Family history of cardiovascular disease, N (%)	11 (64.7%)	119 (59.2%)	0.657
Diabetes, N (%)	6 (35.3%)	25 (12.4%)	**0.010**
Hypercholesterolemia, N (%)	6 (35.3%)	51 (25.4%)	0.371
Hypertension, N (%)	12 (70.6%)	110 (54.7%)	0.206
Current smoker, N (%)	2 (11.8%)	38 (18.9%)	0.465
Angina pectoris, N (%)	4 (23.5%)	92 (44%)	0.076
Metabolic syndrome, N (%)	7 (43.8%)	25 (13.0%)	**0.001**
EAT attenuation (HU), mean (SD)	-98.5 ± 11.8	-101.7 ± 13.0	0.347
NAFLD	5 (29.4%)	52 (26.0%)	0.759
CACS (au) = 0	2 (11.8%)	68 (33.8%)	0.063
CACS (au), median (IQR)	101.0 (4.7-569.0)	27.3 (0-391.8)	0.241
Calcifications in proximal thoracic aorta, median (IQR)	196.4 (12.3-759.5)	14.3 (0-244.6)	**0.023**
Calcifications in aortic root, median (IQR)	146.8 (8.3-758.0)	1.8 (0-175.0)	**0.013**
Calcifications in ascending aorta, median (IQR)	2.1 (0-35.2)	0.0 (0-3.9)	0.109
Myocardial infarction, N (%)	1 (6.0%)	12 (6.0%)	0.988

1 BMI = body mass index; CACS (au) = Agatson coronary artery calcification score; DISH = diffuse idiopathic skeletal hyperostosis (DISH); EAT = epicardial adipose tissue; f/m = female/male; IQR = interquartile range; N = number, NAFLD = nonalcoholic fatty liver disease; SD = standard deviation

**FIGURE 1.**
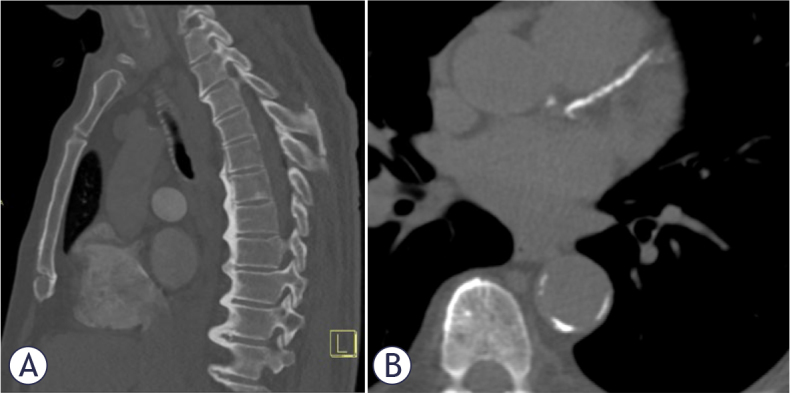
A 68-year old male with diffuse idiopathic skeletal hyperostosis (DISH) coronary artery calcification score (CACS) > 400. **(A)** Typical appearance of DISH in thoracic spine, sagittal plane. **(B)** Calcifications in LAD = left anterior descending artery

Additionally, in subjects with DISH a significantly higher BMI was noted (32.8 *νs*. 28.9) and more diabetes (35.3% *νs*. 12.4%). Figure 1 shows an example of a male patient with DISH and abundant calcifications in left anterior descending coronary artery.

Subjects without DISH were about three times more likely to not have coronary artery calcifications compared to subjects with DISH (33.8% *νs*. 11.0%). In subjects with a CACS > 400, DISH was present in 13.3%, while in subjects with CACS = 0 DISH was present in 2.8% (Table 2).

**TABLE 2.** Prevalence of diffuse idiopathic skeletal hyperostosis (DISH) among Agatson coronary artery calcification score (CACS) categories

	CACS = 0 (N = 70)	CACS > 0 and < 100 (N = 62)	CACS = 100–400 (N = 33)	CACS > 400 (N = 53)
**DISH**	2.8%	10.3%	6.6%	13.3%
**No DISH**	97.2%	89.7%	93.4%	86.7%

Associations between EAT attenuation, cardiovascular risk factors and CT parameters are depicted in Table 3. There is a significant correlation between EAT attenuation and BMI (rho = 0.243, p < 0.001), CACS (rho = 0.256, p < 0.001) and calcifications in ascending aorta (rho = 0.052, p = 0.011), as well as significant association between EAT attenuation and gender (p < 0.001) and NAFLD (p = 0.022).

**TABLE 3.** Association of epicardial adipose tissue (EAT) attenuation with conventional coronary artery disease (CAD) risk factors and CT parameters

Variable	EAT attenuation (HU)	p-value
Gender	M - 98.3 ± 11.3 F - 105.4 ± 13.6	**< 0.001**
NAFLD	Y - 98.3 ± 12.8 N - 102.7 ± 12.8	**0.022**
Family history of cardiovascular disease	Y - 100.1 ± 12.8 N - 104.5 ± 14.1	0.261
Diabetes	Y - 104.2 ± 14.3 N - 101.3 ± 13.3	0.883
Hypercholesterolemia	Y - 99.4 ± 13.5 N - 102.6 ± 13.3	0.402
Hypertension	Y - 99.2 ± 12.4 N - 105.2 ± 14.1	0.129
Smoking	Y - 97.8 ± 13.3 N - 102.9 ± 13.3	0.361
Regular physical activity	Y - 101.6 ± 12.8 N - 101.9 ± 14.4	0.653
	**Correlation coefficient**	
CACS (Agatson)	0.306	**< 0.001**
CACS per vessel		
LM	0.159	**0.018**
LAD	0.247	**< 0.001**
LCX	0.269	**< 0.001**
RCA	0.289	**< 0.001**
Calcifications in proximal thoracic aorta	0.110	0.103
Calcifications in aortic root	0.082	0.226
Calcifications in ascending aorta	0.172	**0.011**
Age	0.006	0.834
BMI	0.243	**< 0.001**

1 BMI = body mass index; CACS = coronary artery calcification score; f = female; HU = Hounsfield units; LAD = left anterior descending artery; LCX = left circumflex artery; m = male; LM = left main coronary artery; NAFLD = nonalcoholic fatty liver disease; RCA = right coronary artery

**FIGURE 2.**
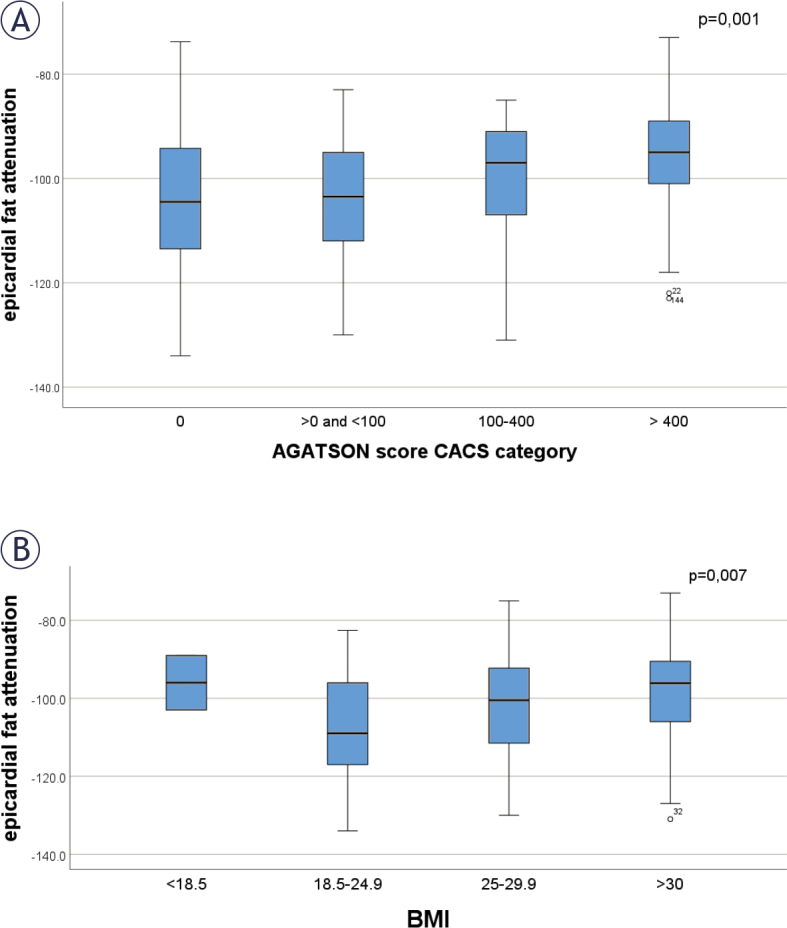
Epicardial fat attenuation in subjects with **(A)** different coronary artery calcium score and **(B)** different body mass index (BMI) categories. Data are presented as box plots, where boxes represent the interquartile range (IQR), the lines within the box represent the median, and the lines outside the boxes represent the upper quartile plus 1.5 times IQR or the lower quartile minus 1.5 times the IQR. CACS = coronary artery calcification score

Figure 2A shows EAT attenuation for patients with different Agatson score CACS category. Mean EAT attenuation was lower in patients with CACS = 0 than in patients with CACS > 400 (-103.7 ± 13.8 HU *νs*. -95.9 ± 11.3 HU [p < 0.001]), also in patients with CACS > 0 and < 100 the mean EAT attenuation was lower than in patients with CACS > 400 (-104.5 ± 12.2 HU *νs*. -95.9 ± 11.3 HU (p < 0.001)).

Group-wise comparisons between BMI categories showed significant differences in EAT attenuation (p = 0.007), as shown in Figure 2B. In patients with BMI < 18.5 EAT attenuation was -96.0 ± 9.9 HU (there were only two patients in this group). In patients with BMI 18.5–24.9 -107.1 ± 13.9 HU, with BMI between 25 and 29.9 -102.1 ± 13.2 HU and in patients with BMI > 30 -98.4 ± 11.6 HU.

On univariate analysis, it was observed that age (p = 0.032), BMI (p = 0.005) and diabetes (p = 0.015) were found to be significantly associated with the presence of DISH (Table 4). In multiple logistic regression model age and BMI were found to be significantly associated with the presence of DISH, odds ratio (OR) 1.060, p = 0.029 and OR 1.132, p = 0.009.

**TABLE 4.** Univariate logistic regression analysis with diffuse idiopathic skeletal hyperostosis (DISH) status as the dependent factor

Variable	units	OR	p-value
Age	+ 1 year	1.055	**0.032**
Gender	Male *vs*. female	2.307	0.129
BMI	+ 1 kg/m^2^	1.133	**0.005**
Diabetes	Present *vs*. absent	3.840	**0.015**
Hypertension	Present *vs*. absent	1.985	0.213
Hypercholesterolemia	Present *vs*. absent	0.623	0.375
Smoking	Present *vs*. absent	1.748	0.470

1 BMI = body mass index; OR = odds ratio

In the multinomial multivariate logistic regression analysis with the different CACS categories as outcome and those without coronary artery calcifications (CACS = 0) as a reference category, gender, age, hypertension and epicardial fat attenuation were found to be significantly associated with the increasing CAC strata (Table 5). Male gender has a 16.786 time greater odds of having CACS > 400 than female gender, compared to subjects with CACS = 0 (p < 0.001). Subjects with hypertension have a 5.423 times greater odds of having CACS > 400 than subjects without hypertension, compared to subjects with CACS = 0 (p < 0.001). There is a 1.227-fold increase in the likelihood of having CACS > 400 with every additional year of age, compared to subjects with CACS = 0 (p < 0.001). Every additional unit of EAT attenuation (HU) increases the odds of having CACS > 400 by 1.052 times when compared to subjects with CACS = 0 (p = 0.022). DISH, smoking status, diabetes, hypercholesterolemia and metabolic syndrome were excluded from the model, since they did not meet the criteria (p < 0.1).

**TABLE 5.** Multinomial multivariate logistic regression analysis on the association of diffuse idiopathic skeletal hyperostosis (DISH) and coronary artery calcification score (CACS) category

CACS category	gender		age		hypertension		EAT attenuation	
	OR	p-value	OR	p-value	OR	p-value	OR	p-value
>0 and <100	3.515	0.008	1.087	<0.001	3.956	0.001	0.980	0.225
100-400	7.583	<0.001	1.156	<0.001	5.023	0.003	1.005	0.804
> 400	16.786	<0.001	1.227	<0.001	5.423	0.001	1.052	0.022

1 OR = odds ratio

1 Coronary artery calcifications (CAC) category is the outcome compared to the subjects without CAC (CACS = 0) as reference category

## Discussion

Despite the fact that DISH is a common condition, epidemiology of the disease in Slovenia is unknown. The overall prevalence of DISH in our cohort was 7.8% (10.4% in men and 4.8% in women). Our results are consistent with the literature, varying from 3.8% in China^24^ to 30.8% in Pakistan^25^, 7.8% in Iceland26 and 12.8% in Italy.^5^ The differences can to some extent be explained by the differences in study population, diagnostic criteria and variety of imaging methods used – chest x-ray or CT scan, whole-spine scans or partial (chest) scans.^26^ Prevalence of DISH increases with age and male to female prevalence ratio is 2:1.^27^ In the current study, subjects with DISH were significantly older than patients without DISH, however, the logistic analysis confirmed ageing to influence the prevalence of DISH significantly.

Previous studies reported higher BMI in patients with DISH than in those without DISH.^26–28^ Also, various metabolic variables are associated with DISH, in particular obesity and type 2 diabetes mellitus.^29,30^ Several paleopathological studies showed that the prevalence of DISH varied significantly between groups of different social standing, with speculation that the upper socioeconomic status groups were excessively nourished, with likely increased incidence of obesity, in comparison with the individuals with lower social status.^29^ In the present study, diabetes and BMI significantly affected the prevalence of DISH in logistic regression analysis. Insulin, a peptide that promotes bone development, is raised in diabetes. It is speculated, that in patients with diabetes, insulin can promote the new bone growth and there by excess bone formation.^31^ Chondrocytes and periosteal mesenchymal cells inside the enthesis can proliferate under the impact of several factors (i.e. insulin, transforming growth factor-β1,…) to form osteoblasts, fibroblasts and myoblasts. Furthermore, different metabolic agents (i.e. insulin, insulin-like growth factor 1,…) have the potential to induce bone formation by stimulating the proliferation of chondrocytes, fibroblasts and osteoblasts.^29^ Increased rates of obesity in DISH patients may indicate that certain adipokines have a role in the disorder’s development. Several of these fat-derived hormones (i.e. leptin) have an association with bone metabolism growth.^3^ Obesity related chronic inflammation with proinflammatory cytokines such as IL-6, TNF-α etc. could contribute to the formation of calcifications, as discs and ligaments of the spine may have receptors for them. Leptin causes chondrocytes to release more chondrocyte degradation mediators and promotes the proliferation of intervertebral disc cells. Leptin stimulates the inflammatory response by raising IL-6, which causes ligamentum flavum hypertrophy and fibrosis.^32^ In this study, subjects with DISH had a higher prevalence of metabolic syndrome and NAFLD than those without DISH. The prevalence of NAFLD and metabolic syndrome rises with obesity; and NAFLD is considered as both, a cause and a result of metabolic syndrome. It is widely documented that NAFLD increases the risk of development of atherosclerosis and cardiac events. Studies showed that NAFLD diagnosed on non-contrast CT to be a strong predictor of MACE (major adverse cardiovascular events) at 14-year follow-up.^22^ We found no correlation between NAFLD and DISH, however there is a significant association between EAT attenuation and NAFLD.

Our study’s findings support earlier research suggesting that DISH is linked to a greater extent of calcifications in blood vessels.^3,9,33^ Indeed, we observed an increase of DISH prevalence across CACS categories. CACS was higher in subjects with DISH compared to the non-DISH group, but the association did not perseverate on multivariate analysis, similar as in previous studies.^34^ It is hypothesized that subjects with DISH may be prone to form calcifications in arteries and in aortic valve, amongst other locations, however, our data point to a more nuanced, maybe non-causal link between CAD and DISH.

The relationship between EAT volume and attenuation, coronary artery plaque load, and coronary artery disease is widely recognized in the literature. In our study EAT attenuation was significantly higher in subjects with CACS > 400 compared to subjects with CACS = 0. Higher EAT attenuation might reflect inflammation in epicardial fat, which was described in patients with acute coronary syndrome.^35^ EAT also increases with vascularization and higher amount of mitochondria and decreases with fatty acids overload.^19^ Statins also decrease EAT attenuation over time, via reducing metabolic activity within the EAT by reducing vascularity, cellularity and inflammation^15^, therefore, an influence of therapy with statins might have influenced the observed EAT attenuation. Among patients with coronary artery disease having open heart surgery, an increase in pro-inflammatory mediators and cytokines in the EAT was reported, as EAT regulates local inflammation in the immediate vicinity of the coronaries.^36^ In our study, chronic, low-grade inflammation might be a significant pathophysiologic connection between DISH, NAFLD, EAT, and CAC. However, to further understand the underlying processes, more research should be conducted correlating EAT attenuation to local and systemic metabolic and inflammatory mechanisms.

The limitations of the current study include its small sample size, the possibility of selection bias due to the inclusion of many individuals with medical disorders, and a cross-sectional design of the study, as a result of which, the possible impact of DISH on mortality cannot be assessed.

To understand the mechanism connecting DISH and coronary artery calcification a multidisciplinary approach that investigates inflammatory, metabolic, genetic, molecular, and environmental factors is required. Future research needs to focus on elucidating common signaling pathways and risk factors that underlie both conditions, employing a combination of molecular, imaging, genetic, and clinical methodologies, with prospective studies and clinical trials, to enhance our comprehension of the fundamental mechanisms.

## Conclusions

There was no independent relationship identified between the prevalence of DISH and CACS. The specific processes that lead to new bone development in DISH patients, particularly in entheses, still remain unclear.
